# Performance comparison of next generation sequencing analysis pipelines for HIV-1 drug resistance testing

**DOI:** 10.1038/s41598-020-58544-z

**Published:** 2020-01-31

**Authors:** Emma R. Lee, Neil Parkin, Cheryl Jennings, Chanson J. Brumme, Eric Enns, Maria Casadellà, Mark Howison, Mia Coetzer, Santiago Avila-Rios, Rupert Capina, Eric Marinier, Gary Van Domselaar, Marc Noguera-Julian, Don Kirkby, Jeff Knaggs, Richard Harrigan, Miguel Quiñones-Mateu, Roger Paredes, Rami Kantor, Paul Sandstrom, Hezhao Ji

**Affiliations:** 1 National HIV and Retrovirology Laboratories, National Microbiology Laboratory at JC Wilt Infectious Diseases Research Centre, Public Health Agency of Canada, Winnipeg, Manitoba Canada; 2 Data First Consulting Inc., Belmont, California USA; 3 Virology Quality Assurance Program, Rush Medical College, Chicago, USA; 4 British Columbia Center for Excellence in HIV/AIDS, Vancouver, British Columbia Canada; 5 Division of Infectious Diseases, Faculty of Medicine, University of British Columbia, Vancouver, British Columbia, Canada; 6 Bioinformatics Core at the National Microbiology Laboratory, Public Health Agency of Canada, Winnipeg, Manitoba Canada; 7 IrsiCaixa AIDS Research Institute, Badalona, Catalonia Spain; 8 Infectious Diseases Service, Hospital Germans Trias, Badalona, Catalonia Spain; 9 Research Improving People’s Lives, Providence, Rhode Island USA; 10 Centre for Research in Infectious Diseases, National Institute of Respiratory Diseases, Mexico City, Mexico; 11 Department of Medical Microbiology and Infectious Diseases, University of Manitoba, Winnipeg, Manitoba Canada; 12 Division of AIDS, Department of Medicine, University of British Columbia, Vancouver, Canada; 13 Department of Microbiology and Immunology, University of Otago, Dunedin, New Zealand; 14 Division of Infectious Diseases, Brown University Alpert Medical School, Providence, Rhode Island USA

**Keywords:** Data processing, Next-generation sequencing

## Abstract

Next generation sequencing (NGS) is a trending new standard for genotypic HIV-1 drug resistance (HIVDR) testing. Many NGS HIVDR data analysis pipelines have been independently developed, each with variable outputs and data management protocols. Standardization of such analytical methods and comparison of available pipelines are lacking, yet may impact subsequent HIVDR interpretation and other downstream applications. Here we compared the performance of five NGS HIVDR pipelines using proficiency panel samples from NIAID Virology Quality Assurance (VQA) program. Ten VQA panel specimens were genotyped by each of six international laboratories using their own in-house NGS assays. Raw NGS data were then processed using each of the five different pipelines including HyDRA, MiCall, PASeq, Hivmmer and DEEPGEN. All pipelines detected amino acid variants (AAVs) at full range of frequencies (1~100%) and demonstrated good linearity as compared to the reference frequency values. While the sensitivity in detecting low abundance AAVs, with frequencies between 1~20%, is less a concern for all pipelines, their specificity dramatically decreased at AAV frequencies <2%, suggesting that 2% threshold may be a more reliable reporting threshold for ensured specificity in AAV calling and reporting. More variations were observed among the pipelines when low abundance AAVs are concerned, likely due to differences in their NGS read quality control strategies. Findings from this study highlight the need for standardized strategies for NGS HIVDR data analysis, especially for the detection of minority HIVDR variants.

## Introduction

Genotypic HIV drug resistance (HIVDR) testing not only guides effective clinical care of HIV-infected patients but also serves to provide surveillance of transmitted HIVDR in the population. Treatment guidelines in resource-permitted settings advocate the use of HIVDR monitoring both prior to ART initiation and when treatment failure is suspected^1,2^. There is increasing evidence showing that the presence of minority resistance variants (MRV) in the HIV quasispecies (i.e., a swarm of highly-related but genotypically different viral variants) may be clinically significant and increase the risk of virological failure, impair immune recovery, lead to accumulation of drug resistance, increase risk of treatment switches and death^3–8^. A nationwide study in Mexico focusing on pretreatment drug resistance (PDR) found that lowering the detection threshold for PDR to 5% versus the conventional 20% improved the ability to identify patients with virological failure^6^. In addition, a European wide study found that pre-existing minority drug-resistant HIV-1 variants more than doubled the risk of virological failure to first-line NNRTI-based ART^9^. A more recent African study also reported similar findings, suggesting lowering the threshold below 20% improved the ability to identify patients who were likely to have virological failure^10^.

The conventional methodology used for HIVDR testing is Sanger sequencing. This method of population-based sequencing generates a single consensus sequence at a 20% threshold which can be analyzed for drug resistant mutations (DRMs). The main drawback of Sanger sequencing is its inability to reliably detect MRV below 20%^11–14^. In contrast, next generation sequencing (NGS) technologies have exceptional resolution and sensitivity for MRV identification^13,15,16^. Other advantages of NGS include improved time efficiency, increased scalability and a reduction in cost when batched specimens are being processed^17–19^. There have been several studies showing that NGS-based HIVDR testing is highly concordant to Sanger sequencing at a 20% threshold and therefore many labs are now transitioning to NGS^17,20–25^. However, several issues still need to be addressed including standardization of NGS-based HIVDR testing protocols and subsequent data processing and reporting, both of which may benefit from improved automation to minimize artificial errors.

As with all molecular laboratory tests, NGS-based HIVDR assays must undergo external quality assessment, and proficiency testing (PT) is a vital component of laboratory quality management. Typically, PT is common for wet-bench procedures and usually includes the entire assay process^26^. Indeed for Sanger-based HIVDR assays, the Virology Quality Assurance (VQA) programs from the NIAID, USA, distributes proficiency panels where the performance of the lab and its assay, including data interpretation, is assessed^27^. FDA-approved kits such as ViroSeq^TM^ and associated bioinformatics software used to analyze Sanger sequencing data such as RECall have been validated as part of Laboratory-Developed Tests^28^. The Sanger sequences are then analyzed by drug resistance interpretation algorithms such as the Stanford HIV Drug Resistance Database^29^. The standardization of NGS-based HIVDR assays is more complex and it includes three main steps: (1) wet-lab steps to generate PCR amplicons that cover the *pol* region and prepare libraries; (2) NGS platforms; and (3) bioinformatics pipelines which convert NGS data into user-interpretable HIVDR results^13,15,30^. Several bioinformatical pipelines have been independently developed to address the needs for automated NGS-based HIVDR genotyping^25,31–39^. We recently published guidelines on the standards for bioinformatics analysis and reporting conventions for HIVDR research and clinical purposes in the “Winnipeg Consensus”. Several recommendations emerged from this meeting covering standards and best practices for (1) NGS read quality control (QC)/quality assurance (QA); (2) NGS read alignment and reference mapping; (3) HIV variant calling and variant QC; (4) NGS HIVDR interpretation and reporting; and (5) general analysis data management^40^. Yet such recommendations remain to be fully implemented in the currently available pipelines and those to be developed.

To determine whether the NGS-based HIVDR data analysis pipelines are concordant, we compared the performance of five commonly-applied NGS HIVDR pipelines including, HyDRA^25^, MiCall^38^, PASeq.^36^, Hivmmer^39^ and DEEPGEN^37^ (see Methods) for HIV amino acid variant (AAV) detection and quantification. AAVs were reported as any amino acid differences from the HIV-1 reference sequence HXB2 or equivalent in the examined genomic fragments. All pipelines are freely available with the exception of DEEPGEN. Assessment parameters included the linear range for AAV frequency measurements, analytical sensitivity and specificity, and variation of detected AAV frequencies. All five pipelines successfully processed NGS data; however, differences in reporting AAV frequencies, especially when they occur at low frequencies support the need to standardize the processing steps in the pipelines, particularly in the area of quality control criteria.

## Methods

### Study sites

The six clinical laboratories that participated in this study included the National HIV and Retrovirology Laboratory (NHRL) at JC Wilt Infectious Disease Center, Winnipeg, Canada; BC Center for Excellence in HIV/AIDS (BC-CfE), Vancouver, Canada; Division of Infectious Diseases, Brown University (BU), Alpert Medical School, Providence, USA; IrsiCaixa AIDS Research Institute, Badalona, Spain; Center for Research in Infectious Diseases (CIENI), National Institute of Respiratory Diseases, Mexico City, Mexico; and Departments of Pathology and Medicine, Case Western Reserve University (CWRU), Cleveland, USA. Three of the laboratories are members of the WHO Global HIV Drug Resistant Network and currently participate in the NIAID VQA program for Sanger-based PT (NHRL, BC-CfE and CIENI).

### Sample processing, library preparation and NGS

A total of ten PT specimens (HIV positive plasma) from two VQA panels, each containing five specimens, were distributed by the NIAID VQA program to each of the six participating laboratories. Each laboratory used their own in-house wet lab methods to extract HIV RNA, obtain PCR amplicons covering the HIV-1 *pol* gene regions targeted in routine HIVDR genotyping (protease (PR), reverse transcriptase (RT), integrase (IN)), and prepare NGS libraries which were subsequently sequenced on either the Illumina MiSeq or Ion Torrent platforms.

### NGS FASTQ submission and pipeline processing

**Figure 1:**
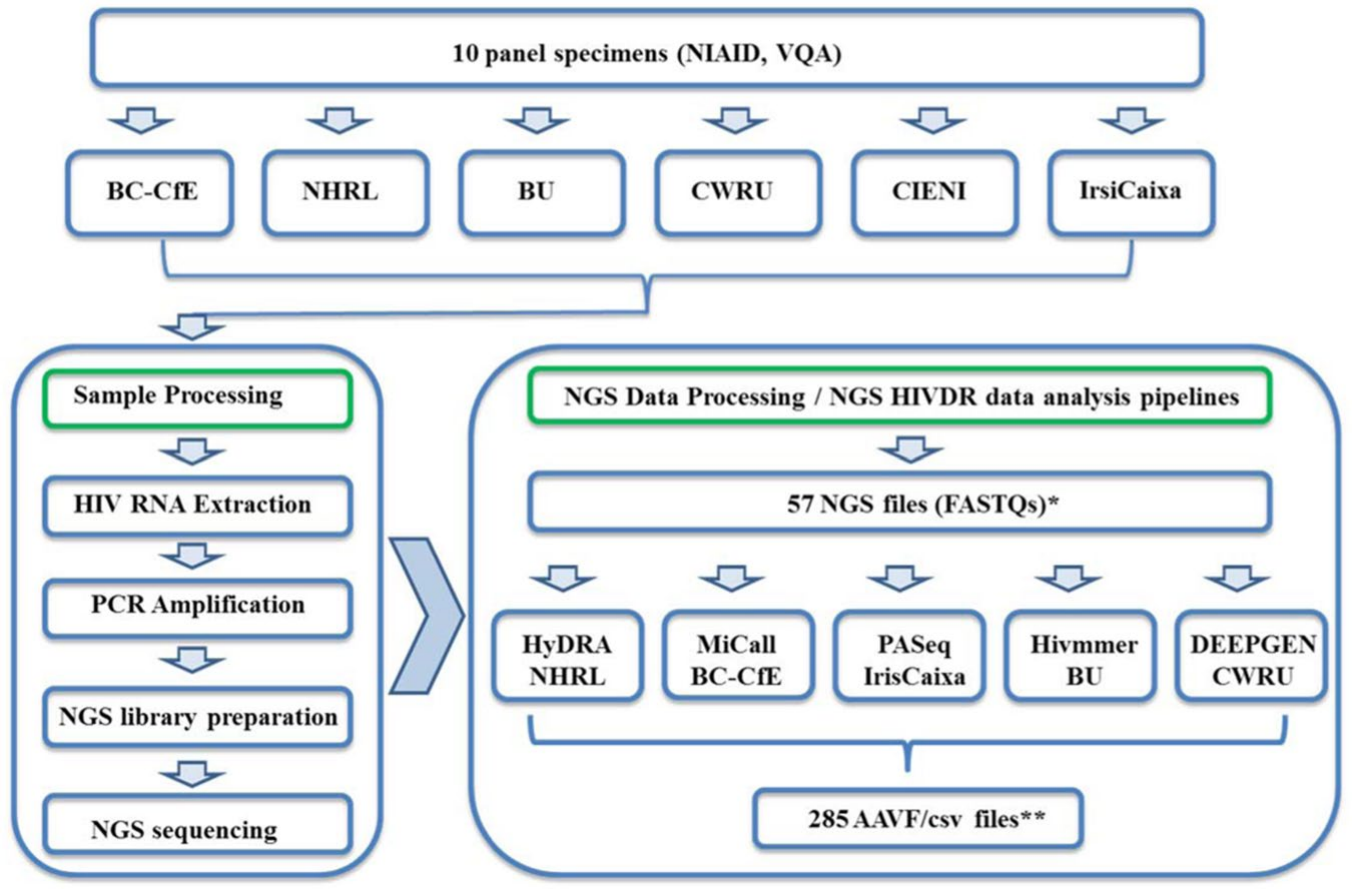
Comparison of NGS HIVDR data analysis pipelines workflow. Abbreviations: NIAID, National Institute of Allergy and Infectious Diseases; VQA, Virology Quality Assurance; BC-CfE, British Columbia Center for Excellence in HIV/AIDS; NHRL, National HIV and Retrovirology Laboratories; BU, Brown University; CWRU, Case Western Reserve University; CIENI, Center for Research in Infectious Diseases; IrisCaxia, AIDS Research Institute. *There are only 57 instead of 60 FASTQ files because 1 lab only processed 7 samples instead of 10. ** Each sample’s pipeline result (AAVF/csv) from each lab was compared, and subsequently all analyses were combined.

Each lab submitted its raw NGS data (in FASTQ format) for each panel specimen to a central location. One lab only successfully processed 7 panel samples so the total number of FASTQ data sets was 57, not 60. The FASTQ files were then processed separately by each pipeline including HyDRA (NHRL), MiCall (BC-CfE), PASeq (IrsiCaixa), Hivmmer (BU), and DEEPGEN (CWRU) (Table 1). All analyses were performed by the developers of each pipeline using default NGS read quality assurance and reference mapping settings for ensured consistency. The AAV frequency outputs (AAVF/csv files) from each pipeline were then uploaded to the central location. The outputs or AAVF/csv files from each pipeline contained all identified AAVs and their frequencies, regardless of their HIVDR relevance, and were compared on a per sample per lab basis where all comparisons were subsequently combined (Fig. 1).

**Table 1:** Comparison of pipelines for automated NGS-based HIVDR data analysis.

	MiCall^38^	HyDRA^25^	PASeq.^36^	Hivmmer^39^	DEEPGEN^37^
URL	https://github.com/cfe-lab/MiCall	https://hydra.canada.ca	https://www.paseq.org	https://github.com/kantorlab/hivmmer	N/A
Bioinformatic IT needs	No	No	No	Yes	N/A
Compatible NGS Platform	Illumina, Ion Torrent	Illumina, Ion Torrent	Illumina, Ion Torrent	Illumina, Ion Torrent	Illumina, Ion Torrent
Web Interface	Yes	Yes	Yes	No	No
Designed for HIVDR	Yes	Yes	Yes	Yes	Yes
Ref Database	HIVdb	HIVdb	HIVdb	HIVdb	HIVdb
Output (aa)	csv	AAVF	csv	AAVF/csv	csv

### Pipeline analytical comparison

To conduct a meaningful comparison of the pipelines, ground truth about present AAVs and their exact frequencies is required, against which further assessment and comparisons could be made. Since none of the tested VQA specimens had been fully characterized for AAVs they are harboring (presence and frequency), such ground truth had to be established based on the data we obtained from the group. Notably, this is the first attempt of such a pipeline comparison, and therefore we had to figure out an innovative way to define which AAV is “truly” present and what reference value could be used as its expected frequency.

The analytical comparison of the pipelines in this study was based on the exclusive inclusion of AAVs being detected at each position by at least four of the five pipelines and at a median frequency threshold of ≥1%, an AAV frequency cut-off for reporting. The median frequency is the midpoint value among the frequency readouts from all pipelines that detect the specific AAV, and it was considered in this study as the “expected” frequency value of the specific AAV. These constitute the basis for the subsequent pipeline performance assessment including (1) linear range, (2) analytical sensitivity, (3) analytical specificity, and (4) variation of the detected AAV frequencies.

The *linear range* of an assay was determined using linear regression analysis where all qualified AAVs and their reported frequencies from all pipelines were analyzed against their expected (median) frequency values. It represents the range of AAV frequencies at a median threshold of ≥1% (1 ~100%) within which linear correlation could be achievable between the expected and observed values.

*Analytical sensitivity* of an assay for AAV detection refers to its ability to detect an AAV when it is present. Based on the median frequency readouts from the pipelines, all qualified AAVs were grouped at frequency thresholds of ≥1%, ≥2%, ≥5%, ≥10%, ≥15% and ≥20%. The *analytical sensitivity* of each pipeline was then calculated for AAVs at all assessed frequency thresholds using the following equation:

1 $${\rm{Analytical}}\,{\rm{sensitivity}}=(1-\text{false} \mbox{-} \text{negative}\,{\rm{rate}})\times 100$$

*where: false-negative rate* = *number of missing AAVs/total number of AAVs*

*Analytical specificity* of an assay for AAV detection refers to its ability of not detecting an AAV when it is absent. The *analytical specificity* of each pipeline was calculated for qualified AAVs at all assessed frequency thresholds (≥1%, ≥2%, ≥5%, ≥10%, ≥15% and ≥20%) using the following equation:

2 $${\rm{Analytical}}\,{\rm{specificity}}=(1-{\rm{false}} \mbox{-} {\rm{positive}}\,\text{rate})\times 100$$

*where*: *false-positive rate* = *number of extra AAVs/total number of AAVs*

*Variation of the detected AAV frequencies* among the pipelines was assessed by (1) examining the outliers of frequency readouts from individual pipelines as compared to the expected AAV frequencies, and (2) pair-wise pipeline comparison of frequency outputs using the Bland-Altman plot. Bland Altman plots are used to compare the agreement between two different instruments or measurement techniques and allows for the identification of any systematic differences between measurements or outliers. In this study, the Bland-Altman plot is used to compare the agreement between two different NGS-based HIVDR pipelines by plotting the percentage of difference in AAV frequency measurements between the two pipelines. To determine the validity of the frequency of the AAVs produced from each pipeline, we took the median frequency across the five pipelines for each AAV, and determined the percent coefficient of variation (%CV), which was calculated as the percent ratio of the standard deviation to the mean frequency readouts from all pipelines. The %CV shows the extent of variability in relation to the mean of the population and is used to assess the precision of a technique^41^. All AAVs were binned into the following frequency intervals: ≥90%, 70–90%, 50–70%, 30–50%, 20–30%, 10–20%, 2–10% and 1–2%. The %CVs for all AAVs in each specific frequency range were then plotted and the medians and interquartile ranges of %CV were determined. Thresholds for outlier identification were empirically determined to be twice the median %CV for each of the aforementioned AAV frequency intervals and were calculated to be %CV ≤ 1%, ≤3%, ≤5%, ≤7%, ≤10%, ≤12%, ≤20%, and ≤24% for AAV frequency ranges of ≥90%, 70–90%, 50–70%, 30–50%, 20–30%, 10–20%, 2–10% and 1–2% respectively. The discordance between the compared pipelines for all AAVs was determined by comparing the levels of agreement between two pipelines using the Bland-Altman plot^42^. All statistical analysis and plotting were performed using GraphPad Prism 8.2.1.

## Results

The assessment of all AAVs versus HIV DRMs allowed for a much larger data set, and better coverage of variations at various frequency ranges with different genetic context. With our defined criteria, a total of 3479 AAVs were identified and included in the pipeline comparison analysis. These AAVs frequencies spanned the entire frequency range (1~100%). The majority of the AAVs (81.46%) were found at frequencies ≥20% as compared to AAVs found at frequencies <20% (18.54%) (Supplementary Fig. S1).

### Linear range

**Figure 2:**
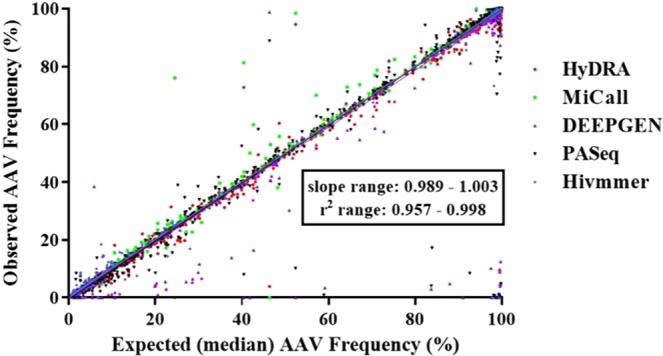
Linearity in AAV frequency measurements between 1% ~ 100% variant frequency.

Linear range reflects the range of AAV frequencies within which linear correlation exists between the detected and the expected values. Linear regression analysis was conducted on 57 data sets from the five pipeline outputs. The range of the *r*^2^ coefficient for all data sets from the five assessed pipelines was 0.957–0.998 and the corresponding slope range was 0.989–1.003, indicating a near-perfect linear correlation between the observed and expected AAV frequencies (Fig. 2, Supplementary Table S1).

### Analytical sensitivity

**Figure 3:**
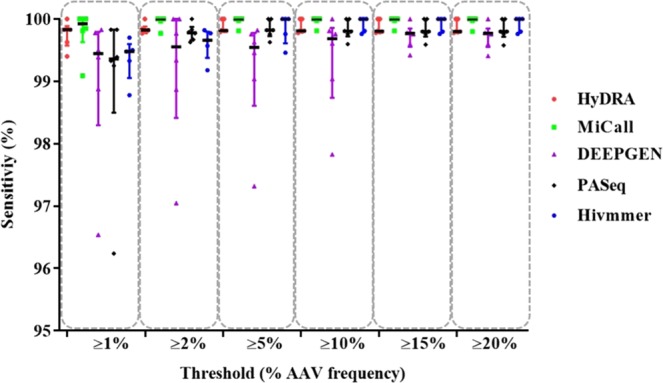
Distribution of sensitivity of NGS HIVDR data analysis pipelines at various AAV frequency thresholds. The scatter plot shows the median and interquartile range for the sensitivity of each pipeline where each point represents one of the six different labs that genotyped VQA specimens at 1%, 2%, 5%, 10%, 15% and 20% thresholds respectively.

Analytical sensitivity of the compared pipelines was calculated by determining the feasibility of an AAV being detected when it exists (see Methods, Eq. 1). It was observed that the sensitivity for all pipelines at each threshold was high where the mean frequencies ranged from 99.36–99.79%, 99.17–99.96%, 99.17–99.97%, 99.34–99.97%, 99.73–99.97%, 99.73–99.97% for AAVs at frequency thresholds of 1%, 2%, 5%, 10%, 15% and 20% respectively (Fig. 3, Supplementary Table S2).

### Analytical specificity

**Figure 4:**
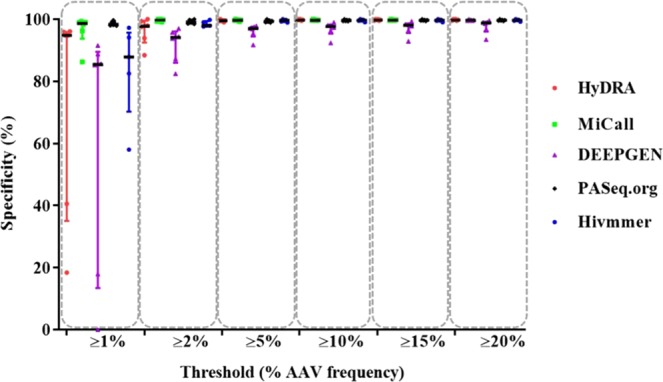
Distribution of specificity of NGS HIVDR data analysis pipelines at various AAV frequency thresholds. The scatter plot shows the median and interquartile range for specificity for each pipeline where each point represents one of the six different labs that genotyped VQA specimens at 1%, 2%, 5%, 10%, 15% and 20% thresholds respectively.

The analytical specificity of the assessed pipelines was calculated by determining the feasibility of an AAV not being detected when it is absent. Our results showed that the mean analytical specificity ranges were 61.56–98.58%, 91.80–99.67%, 96.11–99.68%, 96.87–99.79%, 97.22–99.78% and 97.76–99.78% for AAV frequency thresholds of 1%, 2%, 5%, 10%, 15% and 20% respectively (Fig. 4, Supplementary Table S3). Apart from MiCall and PASeq, the analytical specificity of the pipelines was much lower for AAVs at frequencies <2%. However, out of the six labs that participated in generating NGS FASTQ data sets for pipeline analysis, only two NGS FASTQ data sets resulted in much lower specificity at a 1% threshold. The other four NGS FASTQ data sets resulted in high specificity, for all five pipelines, even at the 1% threshold. (Supplementary Table S3). Specificity increased dramatically at the 2% threshold for all pipelines, suggesting that a 2% threshold may be more reliable than a 1% threshold.

### Variation of detected AAV frequencies amongst the HIVDR NGS pipelines

Bland-Altman analysis, which analyzes the agreement between two compared pipelines, indicated that the percentage of discordances out of the total number of AAVs between two pipelines was similar across all pairwise pipeline comparisons with a range of 0.73–1.46% at 20% threshold, 0.90–1.66% at a 15% threshold, 0.96–2.12% at a 10% threshold, 1.40–2.43% at a 5% threshold, 1.77–2.92% at a 2% threshold, and 2.44–3.62% at a 1% threshold. However, there were differences in the 95% confidence intervals indicating larger discordances between some of the pipelines (Supplementary Fig. S2, Table S4). The number of discordances increased among all pipelines as the AAV frequency threshold decreased with the greatest discordance at the 1% threshold. Similarly, when comparing the difference in discordances between ≥20% and 15% thresholds, 15% and 10% thresholds, 10% and 5% thresholds, 5% and 2% thresholds, and 2% and 1% thresholds, the difference was most significant when comparing the average number of discordances amongst the pipelines at 2% versus 1% threshold (p = 0.0006) (Supplementary Table S5). In agreement with the results found with specificity, the discordance results also suggest that a 2% threshold may be more reliable than a 1% threshold.

**Figure 5:**
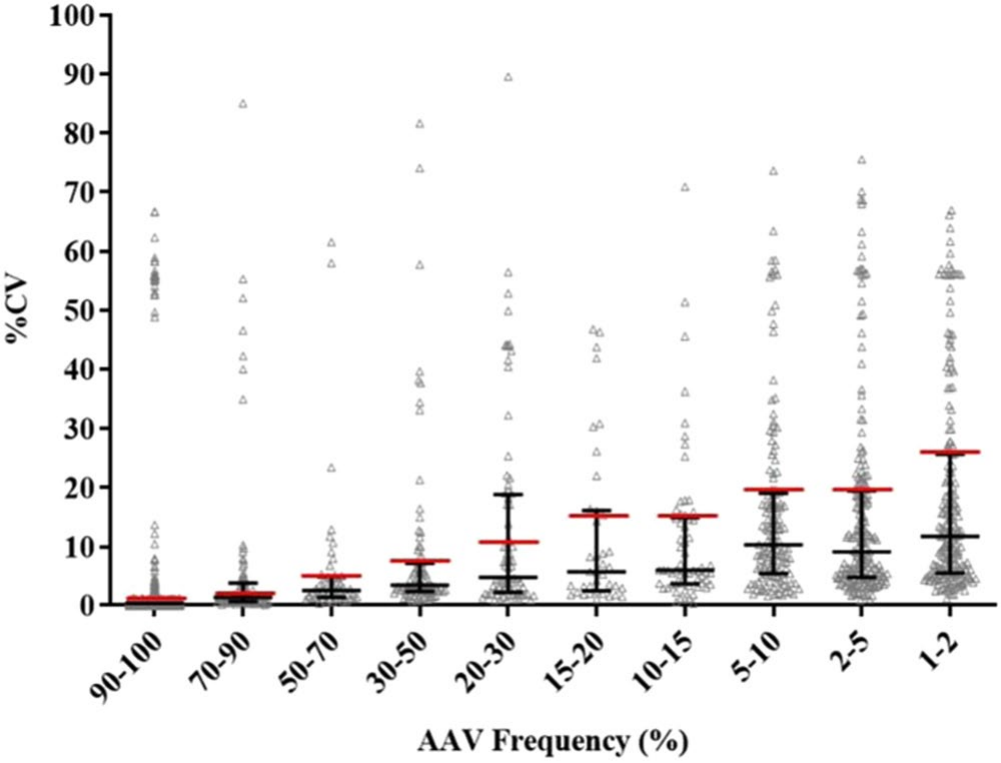
Distribution of %CV measurements of AAV frequencies between 1~100%. The scatter plot shows the median and interquartile range for %CV at AAV frequencies between 1~100%. Thresholds for outliers are shown by the red line and are equal to twice the %CV median for each range (see Methods).

An outlier is an observation that lies an abnormal distance from other values in a random sample from a population^43^. Figure 5 depicts all AAVs at different ranges of frequencies and their %CVs as calculated using the frequency readouts from all five pipelines. As described in the Methods, thresholds for outliers were empirically determined to be twice the median %CV for each defined %AAV frequency range. The detailed outlier counts for all pipelines were summarized in Table 2. In total, 412 outliers were observed across the pipelines. There were more outliers observed at AAV frequencies <20% (n = 214) as compared to AAVs >20% (n = 198) (Table 2, Supplementary Fig. S3). Most of the outliers >20% were generated from DEEPGEN; with this pipeline removed from the analysis, the outliers <20% outnumbered the outliers >20% by nearly 2:1.

**Table 2:** Summary of NGS HIVDR data analysis pipeline outliers.

%AAV Frequency Range	%CV threshold	Number of outliers above %CV threshold*				
		HyDRA	MiCall	DEEPGEN	PASeq	Hivmmer
90-100	≤1	14	2	62	27	12
70-90	≤3	4	3	10	6	4
50-70	≤5	1	2	2	4	2
30-50	≤7	3	2	6	2	3
20-30	≤10	4	0	10	8	5
15-20	≤12	3	0	4	4	5
10-15	≤12	8	1	3	10	5
5-10	≤20	8	1	11	11	11
2-5	≤20	10	8	10	15	20
1-2	≤24	11	11	12	21	11
**Number of outliers** ≥**20%**		**26**	**9**	**90**	**47**	**26**
**Number of outliers** <**20%**		**40**	**21**	**40**	**61**	**52**

*Data from one of the six participating labs was removed from outlier analysis because the results from one pipeline were missing. In this case, there were 47 data sets as opposed to 57 (see Methods). The %CV thresholds for outliers are equal to twice the %CV median for each %AAV frequency range.

Outliers were more commonly encountered in six positions in the *pol* gene— IN D10, IN D288, PR S37, RT D67, RT T200 and RT Q207, where only one variant at position RT D67 results in a DRM (D67N) (Supplementary Table S6, Supplementary Fig. S3). Most pipelines occasionally missed an AAV or reported it at a lower or higher frequency than the median. However, DEEPGEN also reported additional AAVs at positions RT T215, PR S37, RT D67, RT T200 and RT Q207 at ≥5% frequency. Mutations found in IN, specifically D10E and D288N, did not contain any homopolymer regions or inverted repeats, however, these positions are near ends of the IN gene. Mutations in protease and reverse transcriptase specifically PR S37A/D, RT D67N, RT T200A and RT Q207N were in regions containing homopolymers or inverted repeats which may have contributed to NGS errors^44–48^, resulting in missed detections or detection at lowered frequencies.

## Discussion

Transitioning from Sanger to NGS-based HIVDR genotyping requires a substantial methodological overhaul throughout the entire process. Sanger-based HIVDR genotyping reports DRM data as dichotomous (present or absent), whereas NGS-based HIVDR genotyping also reports DRMs as numerical data (relative abundance). This additional information strengthens our ability to assess the clinical impact of a given DRM and to determine and track its overall frequency within a population, which may significantly impact drug regimens and public health approaches to control and reduce HIV transmission^10,14,49–52^. Notably, while many NGS HIVDR data analysis pipelines exist^25,31–39^, their design and implementation was conducted independently by different research groups with little coordination among the developers. Given the complexity of the analysis and the varied approaches adopted by the different development teams, this historical lack of coordination results in uncertainties in the reliability of the data. We undertook this study to compare and evaluate the performance of five popular NGS HIVDR pipelines using several assessment strategies. Instead of restricting our analysis to known DRMs, we chose to analyse all detected AAVs in order to obtain a more fulsome data set.

While DEEPGEN was originally designed for analyzing Ion Torrent data, all the other compared pipelines in this paper were primarily designed for Illumina data processing. Despite this, all pipelines showed excellent correlation in detecting AAVs in a frequency range between 1% and approximately 100%. Linear regression analysis showed a strong correlation between the expected and observed values for all assessed pipelines. In addition, all pipelines showed strong analytical sensitivity and were able to detect most AAVs that were present even at the 1% threshold. This is due to the ability of NGS technologies to sequence viral genomes with extremely high coverage. However, with the exception of PASeq and MiCall, significantly lower analytical specificity was observed at the 1% threshold, where reported AAVs detected by some pipelines, were not detected by other pipelines resulting in false positives. Importantly, the decrease in specificity at the 1% threshold was only found in the pipeline analysis of FASTQs derived from two of the six labs. The remaining labs’ FASTQ data was analyzed with much higher specificity at the 1% threshold for all five pipelines suggesting that perhaps the NGS run from these two labs, was of lower quality compared to NGS runs from the other four labs. In addition, although the inherent error rate of NGS may be below 2% (for some platforms), some NGS-detected variations at frequencies around 1–2% are likely attributable to a combination of uneven sampling, PCR-induced errors, and inconsistent NGS data quality control. In any event, the data from this study shows that increasing the threshold from 1% to 2% dramatically increases the specificity and consequently a 2% threshold may be more reliable than a 1% for NGS-based AAV or HIV DRM identification. An evaluation by *M. Perrier et al*., which compared three NGS alignment algorithms for HIV-1 MRV also suggests a 2% threshold may be more robust than 1%^53^. In their study, they focus exclusively on minority resistant variants with an interest in the concordance of the different algorithms. In contrast, we included all frequencies (1~100%) and all amino acid variants in order to obtain a much larger data set and to evaluate a full range of frequencies. We looked not only at concordance between pipelines, but also specificity, sensitivity, number of outliers and AAV frequency distribution.

To determine whether the pipelines were consistent in reporting AAV frequencies, we compared the frequency outputs for all AAVs from all five pipelines. Typically, outliers are measured by whether the data point is higher or lower than two standard deviations from the mean, or by exceeding the interquartile range (IQR)^43^. Initially, we used scatter plots to highlight the IQR and to visually inspect obvious outliers. We calculated whether the outlier fell outside the upper and lower limits; however, the NGS AAV frequency data did not always conform to IQR analysis, because sometimes the range of AAV frequencies was too broad to identify one or two outliers. Instead, we determined a different method of analysis to identify outliers. As described in Methods, our assessment of pipelines was based on whether the AAV was found in at least four out of the five pipelines at a frequency threshold of 1% or greater. The median frequency of a particular AAV was considered as the “expected value”, against which all observed frequencies for each AAV were compared. Thresholds for outliers were arbitrarily determined to be twice the median %CV for each defined AAV frequency range.

There were several positions in the *pol* gene where outliers were found; most commonly at positions IN D10, IN D288, PR S37, RT D67, RT T200 and RT Q207. This is especially the case for DEEPGEN, which either reported AAVs at a much lower frequency or completely missed detecting AAVs at positions IN D10, PR S37, RT D67, RT T200 and RT Q207 compared to the other pipelines. In addition DEEPGEN also reported additional AAVs at positions RT T215, PR S37, RT D67, RT T200 and RT Q207 at ≥5% frequency, that were not reported by any other pipeline. Both HyDRA and Hivmmer had difficulties detecting variants at IN D288 perhaps because it lies near the stop codon. The other positions all fell in regions that had homopolymers or inverted repeats which may have impacted the ability of the sequencing platform^44–48^ and consequently the ability of the pipeline to detect the AAV or detect it at a lower frequency. With the exception of DEEPGEN, all pipelines had more outliers at frequencies below 20%.

To our knowledge, while all pipelines may accommodate FASTQ data from both platforms each with its platform-specific issues, none of these pipelines offers separate solutions or settings for analyzing data from any specific platform. The unusual outlier distribution for DEEPGEN may be partly due to its design in analyzing Ion Torrent data, whereas all other pipelines were based on Illumina data. The Ion Torrent has been reported to have issues resolving sequences in homopolymer regions due to its ion semiconductor-based sequencing approach^47,48^. In addition, the DEEPGEN pipeline had some instances where the reference sequence became distorted in the integrase region and caused problems in sequence alignment, although the challenge of detecting AAVs using read mapping strategies is an inherent difficulty encountered by all pipelines that adopt this approach. Overall, as the frequency range increased, the %CV threshold decreased meaning that the higher the frequency range, the more stringent the %CV cut-off could be. If we analyzed the data at one consistent %CV threshold (i.e., %CV ≤ 24%), then fewer outliers would be observed above 20% abundance and the majority of the outliers would be below 20%, with the most being observed at 1–2%. While the issue is debatable over what threshold should be applicable for NGS-based HIVDR assays, and when MRV may become clinically relevant, our observations strongly suggest that a 2% threshold is more reliable than a 1% threshold for NGS-based MRV detection and reporting.

Although we found high concordance among the pipelines, and high linearity for expected-versus-observed AAV frequency, we acknowledge that some limitations exist in this study including: (1) wet lab steps were not considered in this study; only NGS data processing and subsequent variant reporting was addressed. All wet labs steps including the initial viral template input, nucleic acid extraction, fraction of nucleic acids used for RT-PCR, efficiency of RT-PCR/PCR, and NGS library preparation still require standardization and likely have a significant impact on variant reporting^13,54^; (2) the majority of the NGS data used for processing came from Illumina technology which is currently the most dominant technology but not the only platform for NGS HIVDR^55^; (3) notably, further work needs to be done to develop a more sophisticated statistical analysis method to identify outliers and better assess the variations among different NGS platforms or pipelines; and (4) the study lacked well-characterized proficiency testing (PT) materials, which limits our capacity to determine the authenticity of any AAV and its exact frequency in the test specimen. While different solutions had been considered in the establishment of our criteria for defining the “true” AAVs and the reference values for their frequencies, its representativeness for the ground truth remains debatable. Development of fully characterized PT panels would allow us to better assess the performance of an NGS assay and/or a pipeline’s ability in detecting AAVs at a known frequency.

Overall, all the assessed pipelines function well in their ability to detect and quantify AAV frequencies although their performance varies. Discrepancies may be due to different NGS platforms, problem areas in the HIV genome or intrinsic drawbacks within the pipelines. Our findings support that a 2% cutoff may be suitable for NGS-based HIVDR assays. Analytical sensitivity, analytical sensitivity and in-depth variation analysis for detected AAVs and their frequencies may assist in the in-depth performance evaluation of an NGS HIVDR assay for quality assessment purposes.

## Supplementary information

Supplementary information.
