# Supplementary material for: Performance comparison of next generation sequencing analysis pipelines for HIV-1 drug resistance testing
Source: Sci Rep. 2020 Jan 31;10:1634. doi: 10.1038/s41598-020-58544-z (PMC6994664; doi:10.1038/s41598-020-58544-z)
Supplement: Supplementary file 1 — Supplementary information. [file 41598_2020_58544_MOESM1_ESM.docx]

**Supplementary Information**

**Performance comparison of next generation sequencing analysis pipelines for HIV-1 drug resistance testing**

Emma R. Lee^1^, Neil Parkin^2^, Cheryl Jennings^3^, Chanson J. Brumme^4,5^, Eric Enns^6^, Maria Casadellà^7^, Mark Howison^9^, Mia Coetzer^14^, Santiago Avila Rios^10^, Rupert Capina^1^, Eric Marinier^6^, Gary Van Domselaar^6,11^, Marc Noguera-Julian^7^, Don Kirby^4,5^, Jeff Knaggs^4,5^, Richard Harrigan^12^, Miguel Quiñones-Mateu^13^, Roger Paredes^7,8^ , Rami Kantor^14^, Paul Sandstrom^1,11^, Hezhao Ji^1,11^ **^§^**

^1^ National HIV and Retrovirology Laboratories, National Microbiology Laboratory at JC Wilt Infectious Diseases Research Centre, Public Health Agency of Canada, Winnipeg, Manitoba, Canada

^2^ Data First Consulting Inc., Belmont, California, USA

^3^ Virology Quality Assurance Program, Rush Medical College, Chicago, USA

^4^ British Columbia Center for Excellence in HIV/AIDS, University of British Columbia, Vancouver, British Columbia, Canada

^5^ Division of Infectious Diseases, Faculty of Medicine, University of British Columbia, Vancouver, Canada

^6^ Bioinformatics Core at the National Microbiology Laboratory, Public Health Agency of Canada, Winnipeg, Manitoba, Canada

^7^ IrsiCaixa AIDS Research Institute, Badalona, Catalonia, Spain

^8^ Infectious Diseases Service, Hospital Germans Trias, Badalona, Catalonia, Spain

^9^ Research Improving People’s Lives, Providence, Rhode Island, USA

^10^ Centre for Research in Infectious Diseases, National Institute of Respiratory Diseases, Mexico City, Mexico

^11^ Department of Medical Microbiology and Infectious Diseases, University of Manitoba, Winnipeg, Manitoba, Canada

^12^ Division of AIDS, Department of Medicine, University of British Columbia, Vancouver, Canada

^13^ Department of Microbiology and Immunology, University of Otago, Dunedin, New Zealand

^14^ Division of Infectious Diseases, Brown University Alpert Medical School, Providence, Rhode Island, USA.

**^§^** Corresponding author at:

National HIV & Retrovirology Laboratories

National Microbiology Laboratory at JC Wilt Infectious Diseases Research Centre,

Public Health Agency of Canada,

Winnipeg, Canada

Tel: 1-204-789-6521

Fax: 1-204-318-2221

Email: hezhao.ji@canada.ca

**Supplemental Data**


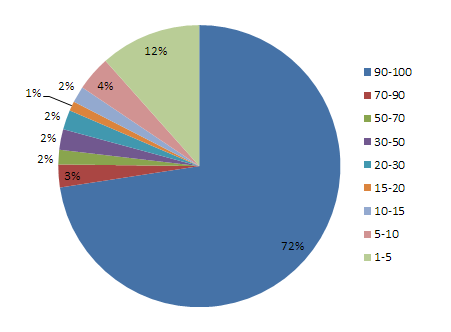


**Supplementary Figure S1.** Distribution of AAV frequencies.

|  | **HyDRA** | **MiCall** | **DEEPGEN** | **PASeq** | **hivmmer** |
| --- | --- | --- | --- | --- | --- |
| **Slope** | 0.9984 ± 0.0014 | 1.001 ± 0.0007 | 0.9892 ± 0.0035 | 1.003 ± 0.0023 | 0.9958 ± 0.0024 |
| **r^2^** | 0.9928 | 0.9981 | 0.9567 | 0.9822 | 0.9917 |

**Supplementary Table S1.** Linear aggression analysis between observed and expected AAV frequencies for each pipeline.

|  |  | **Pipeline** | | | | |
| --- | --- | --- | --- | --- | --- | --- |
| **Source of FASTQ data** | **% threshold** | **HyDRA** | **Micall** | **DEEPGEN** | **PASeq** | **Hivmmer** |
| Brown University | ≥1% | 99.83 | 100 | 99.83 | 99.83 | 99.48 |
| Providence, USA | ≥2% | 99.79 | 100 | 100 | 99.79 | 99.57 |
|  | ≥5% | 99.82 | 100 | 99.82 | 99.82 | 99.46 |
|  | ≥10% | 99.81 | 100 | 99.81 | 99.81 | 99.81 |
|  | ≥15% | 99.8 | 100 | 99.8 | 99.8 | 99.8 |
|  | ≥20% | 99.8 | 100 | 99.8 | 99.8 | 99.8 |
| National Microbiology | ≥1% | 99.85 | 99.09 | 99.39 | 99.39 | 98.78 |
| Laboratory, PHAC | ≥2% | 99.84 | 100 | 99.34 | 99.67 | 99.18 |
| Winnipeg, Canada | ≥5% | 99.81 | 100 | 99.63 | 99.63 | 100 |
|  | ≥10% | 99.8 | 100 | 100 | 99.6 | 100 |
|  | ≥15% | 99.79 | 100 | 100 | 99.59 | 100 |
|  | ≥20% | 99.79 | 100 | 100 | 99.58 | 100 |
| British Columbia | ≥1% | 99.83 | 100 | 99.5 | 99.83 | 99.5 |
| Center for Excellence | ≥2% | 99.83 | 100 | 100 | 99.83 | 99.66 |
| in HIV/AIDS | ≥5% | 100 | 100 | 99.46 | 99.82 | 100 |
| Vancouver, Canada | ≥10% | 100 | 100 | 99.61 | 99.8 | 100 |
|  | ≥15% | 100 | 100 | 99.6 | 99.8 | 100 |
|  | ≥20% | 100 | 100 | 99.6 | 99.8 | 100 |
| Case Western | ≥1% | 99.63 | 99.81 | 98.88 | 99.25 | n/a |
| Reserve University | ≥2% | 99.77 | 99.77 | 98.87 | 100 | n/a |
| Cleveland, USA | ≥5% | 99.81 | 99.81 | 99.04 | 100 | n/a |
|  | ≥10% | 99.81 | 99.81 | 99.04 | 100 | n/a |
|  | ≥15% | 99.81 | 99.81 | 99.42 | 100 | n/a |
|  | ≥20% | 99.8 | 99.8 | 99.41 | 100 | n/a |
| IrisCaxia AIDS | ≥1% | 100 | 100 | 99.78 | 99.33 | 99.33 |
| Research Institute | ≥2% | 100 | 100 | 99.77 | 99.77 | 99.77 |
| Badalona, Spain | ≥5% | 100 | 100 | 99.76 | 99.76 | 99.76 |
|  | ≥10% | 100 | 100 | 99.76 | 99.76 | 99.76 |
|  | ≥15% | 100 | 100 | 99.76 | 99.76 | 99.76 |
|  | ≥20% | 100 | 100 | 99.76 | 99.76 | 99.76 |
| Center for Research | ≥1% | 99.4 | 99.85 | 96.54 | 96.24 | 99.7 |
| in Infectious Diseases | ≥2% | 99.82 | 100 | 97.05 | 99.63 | 99.82 |
| (CIENI) | ≥5% | 99.79 | 100 | 97.32 | 100 | 100 |
| Mexico City, Mexico | ≥10% | 99.78 | 100 | 97.83 | 100 | 100 |
|  | ≥15% | 99.78 | 100 | 99.78 | 100 | 100 |
|  | ≥20% | 99.78 | 100 | 99.78 | 100 | 100 |
|  | mean ± SD ≥1% | 99.76 ± 0.21 | 99.79 ± 0.35 | 98.99 ± 1.25 | 98.98 ± 1.37 | 99.36 ± 0.35 |
|  | mean ± SD ≥2% | 99.84 ± 0.08 | 99.96 ± 0.09 | 99.17 ± 1.13 | 99.78 ± 0.13 | 99.60 ± 0.25 |
|  | mean ± SD ≥5% | 99.87 ± 0.10 | 99.97 ± 0.08 | 99.17 ± 0.95 | 99.84 ± 0.14 | 99.84 ± 0.24 |
|  | mean ± SD ≥10% | 99.87 ± 0.10 | 99.97 ± 0.08 | 99.34 ± 0.81 | 99.83 ± 0.15 | 99.91 ± 0.12 |
|  | mean ± SD ≥15% | 99.86 ± 0.11 | 99.97 ± 0.08 | 99.73 ± 0.20 | 99.83 ± 0.16 | 99.91 ± 0.12 |
|  | mean ± SD ≥20% | 99.86 ± 0.11 | 99.97 ± 0.08 | 99.73 ± 0.20 | 99.82 ± 0.16 | 99.91 ± 0.12 |

**Supplementary Table S2.** The sensitivity of NGS HIVDR data analysis pipelines at various AAV frequency thresholds.

|  |  | **Pipeline** | | | | |
| --- | --- | --- | --- | --- | --- | --- |
| **Source of FASTQ data** | **% threshold** | **HyDRA** | **Micall** | **DEEPGEN** | **PASeq** | **Hivmmer** |
| Brown University | ≥1% | 94.81 | 98.96 | 85.3 | 99.14 | 97.23 |
| Providence, USA | ≥2% | 99.36 | 100 | 95.73 | 99.79 | 99.79 |
|  | ≥5% | 99.64 | 99.64 | 96.93 | 99.46 | 99.64 |
|  | ≥10% | 99.81 | 99.62 | 97.34 | 99.43 | 99.81 |
|  | ≥15% | 99.8 | 99.8 | 97.83 | 99.41 | 99.8 |
|  | ≥20% | 99.8 | 99.8 | 98.6 | 99.4 | 99.8 |
| National Microbiology | ≥1% | 95.89 | 98.33 | 85.69 | 99.7 | 82.5 |
| Laboratory, PHAC | ≥2% | 100 | 100 | 97.05 | 100 | 99.02 |
| Winnipeg, Canada | ≥5% | 100 | 100 | 97.41 | 100 | 100 |
|  | ≥10% | 100 | 100 | 98.81 | 100 | 100 |
|  | ≥15% | 100 | 100 | 99.38 | 100 | 100 |
|  | ≥20% | 100 | 100 | 99.58 | 100 | 100 |
| British Columbia Center | ≥1% | 94.83 | 99 | 88.82 | 98 | 94.16 |
| Center for Excellence | ≥2% | 97.98 | 99.32 | 94.44 | 98.48 | 97.64 |
| In HIV/AIDS | ≥5% | 99.46 | 99.64 | 97.11 | 98.73 | 98.92 |
| Vancouver, Canada | ≥10% | 99.61 | 99.61 | 98.03 | 99.41 | 99.01 |
|  | ≥15% | 99.6 | 99.6 | 98.4 | 99.6 | 99 |
|  | ≥20% | 99.6 | 99.6 | 99 | 99.6 | 99.2 |
| Case Western | ≥1% | 96.08 | 96.26 | 91.59 | 98.51 | n/a |
| Reserve University | ≥2% | 97.51 | 99.1 | 93.88 | 99.1 | n/a |
| Cleveland, USA | ≥5% | 99.43 | 99.23 | 95.4 | 99.62 | n/a |
|  | ≥10% | 100 | 99.62 | 96.34 | 99.81 | n/a |
|  | ≥15% | 100 | 99.61 | 96.51 | 99.81 | n/a |
|  | ≥20% | 100 | 99.61 | 96.85 | 99.8 | n/a |
| IrisCaxia AIDS | ≥1% | 18.43 | 99.33 | 17.98 | 98.2 | 87.87 |
| Research Institute | ≥2% | 88.4 | 99.77 | 87.24 | 98.61 | 97.91 |
| Badalona, Spain | ≥5% | 99.05 | 99.77 | 97.87 | 99.05 | 99.29 |
|  | ≥10% | 99.52 | 99.76 | 98.1 | 99.76 | 99.52 |
|  | ≥15% | 99.52 | 99.76 | 98.31 | 99.76 | 99.52 |
|  | ≥20% | 99.51 | 99.76 | 99.03 | 99.76 | 99.51 |
| Center for Research | ≥1% | 40.6 | 86.32 | 0 | 97.9 | 58.05 |
| In Infectious Diseases | ≥2% | 93.91 | 99.82 | 82.47 | 99.08 | 97.97 |
| (CIENI) | ≥5% | 99.59 | 99.79 | 91.75 | 99.18 | 99.79 |
| Mexico City, Mexico | ≥10% | 99.78 | 99.78 | 92.41 | 99.35 | 99.78 |
|  | ≥15% | 99.78 | 99.78 | 92.91 | 99.56 | 99.78 |
|  | ≥20% | 99.78 | 99.78 | 93.48 | 99.55 | 99.78 |
|  | mean ± SD ≥1% | 73.44 ± 34.74 | 96.37 ± 5.05 | 61.56 ± 41.18 | 98.58 ± 0.71 | 83.96 ± 15.56 |
|  | mean ± SD ≥2% | 96.19 ± 4.37 | 99.67 ± 0.37 | 91.80 ± 5.70 | 99.18 ± 0.61 | 98.47 ± 0.91 |
|  | mean ± SD ≥5% | 99.53 ± 0.31 | 99.68 ± 0.26 | 96.11 ± 2.30 | 99.37 ± 0.40 | 99.53 ± 0.43 |
|  | mean ± SD ≥10% | 99.79 ± 0.20 | 99.73 ± 0.15 | 96.87 ± 2.36 | 99.63 ± 0.27 | 99.62 ± 0.38 |
|  | mean ± SD ≥15% | 99.78 ± 0.20 | 99.76 ± 0.15 | 97.22 ± 2.31 | 99.69 ± 0.21 | 99.62 ± 0.39 |
|  | mean ± SD ≥20% | 99.78 ± 0.20 | 99.76 ± 0.15 | 97.76 ± 2.29 | 99.69 ± 0.21 | 99.66 ± 0.31 |

**Supplementary Table S3.** The specificity of NGS HIVDR data analysis pipelines at various AAV frequency thresholds.


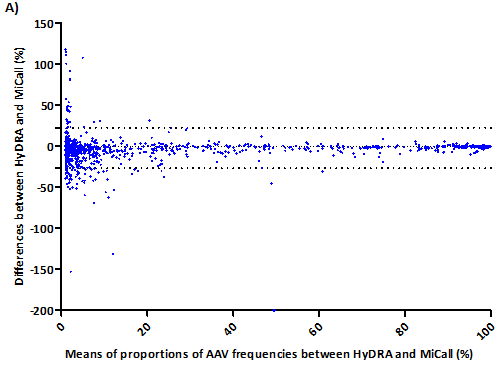

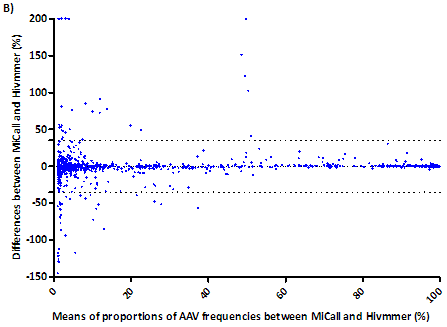


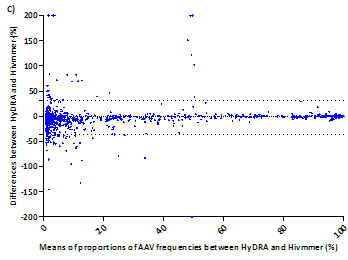

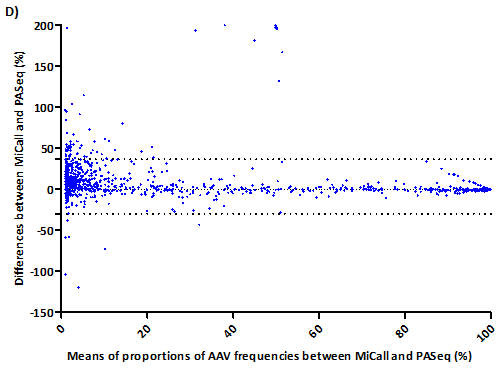


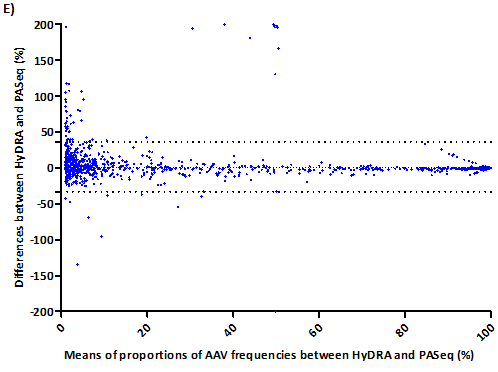

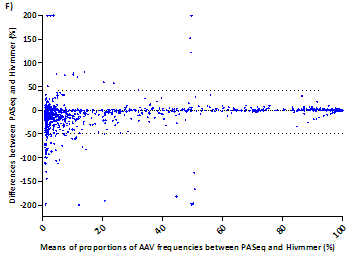


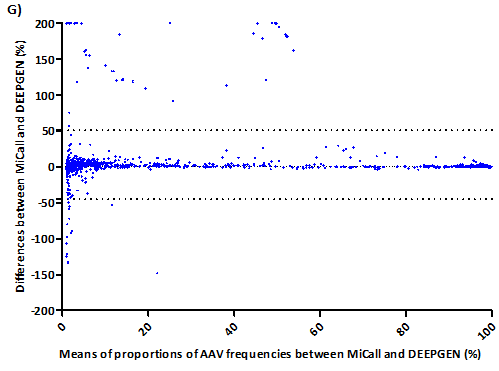

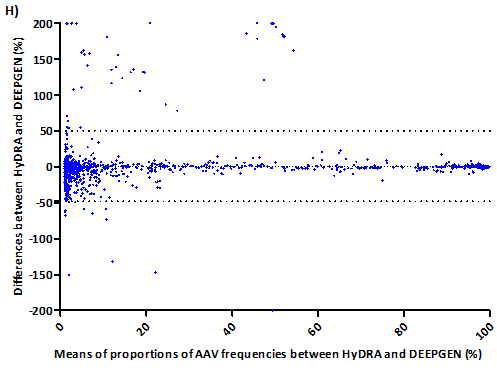


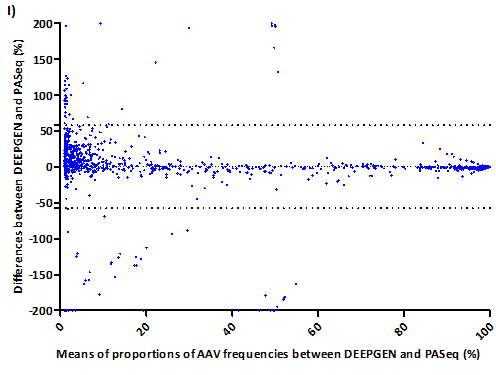

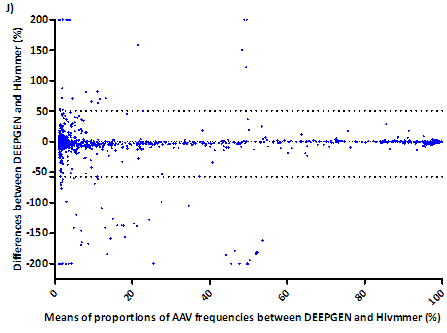


**Supplementary Figure S2.** Comparison of NGS HIVDR data analysis pipelines. The agreement between two different NGS-based HIVDR pipelines was assessed using the Bland-Altman plot which plotted the percentage of difference in AAV frequency measurements (y-axis) between A) HyDRA and MiCall, B) MiCall and Hivmmer, C) HyDRA and Hivmmer, D) MiCall and PASeq, E) HyDRA and PASeq, F) PASeq and Hivmmer, G) MiCall and DEEPGEN, H) HyDRA and DEEPGEN, I) DEEPGEN and PASeq, J) DEEPGEN and Hivmmer and x-axis indicates the mean between the two measurements. The 95% confidence intervals limits of agreement were calculated and upper and lower limits of agreement are depicted by the dotted lines.

| **Analysis pipelines** | **Number of discordances, n (%), 95% confidence intervals** | | | | | |
| --- | --- | --- | --- | --- | --- | --- |
|  | **≥ 20% threshold** | **≥ 15% threshold** | **≥ 10% threshold** | **≥ 5% threshold** | **≥ 2% threshold** | **≥ 1% threshold** |
| HyDRA *versus* MiCall | 22 (0.95%) (-13.55-11.78) | 28 (1.19%) (-13.83-11.91) | 32 (1.33%) (-16.67-14.21) | 56 (2.22%) (-18.22-15.09) | 78 (2.86%) (-21.99-18.04) | 102 (3.43%) (-26.49-22.73) |
| MiCall *versus* Hivmmer | 24 (1.03%) (-15.36-16.50) | 25 (1.06%) (-15.51-16.64) | 37 (1.54%) (-17.67-18.65) | 52 (2.06%) (-18.96-19.72) | 67 (2.46%) (-24.72-26.22) | 83 (2.78%) (-35.46-34.95) |
| HyDRA *versus* Hivmmer | 24 (1.03%) (-20.42-19.78) | 28 (1.20%) (-20.69-19.91) | 44 (1.83%) (-23.94-22.45) | 58 (2.30%) (-25.63-23.42) | 77 (2.84%) (-30.23-27.77) | 101 (3.37%) (-35.92-31.71) |
| MiCall *versus* PASeq | 21 (0.90%) (-25.21-27.19) | 26 (1.11%) (-25.27-27.40) | 36 (1.50%) (-25.78-28.34) | 51 (2.03%) (-26.17-29.78) | 69 (2.54%) (-27.10-32.53) | 85 (2.89%) (-29.68-36.73) |
| HyDRA *versus* PASeq | 17 (0.73%) (-28.45-28.57) | 21 (0.90%) (-28.50-28.70) | 23 (0.96%) (-28.35-28.61) | 35 (1.40%) (-28.92-29.39) | 48 (1.77%) (-29.89-31.16) | 72 (2.44%) (-33.00-36.42) |
| PASeq *versus* Hivmmer | 27 (1.16%) (-29.35-28.63) | 32 (1.37%) (-29.58-28.65) | 43 (1.79%) (-32.53-30.80) | 61 (2.43%) (-34.27-31.49) | 79 (2.92%) (-39.51-35.54) | 108 (3.62%) (-49.21-41.41) |
| MiCall *versus* DEEPGEN | 25 (1.08%) (-34.46-40.23) | 28 (1.19%) (-34.95-41.03) | 37 (1.54%) (-37.07-44.02) | 43 (1.71%) (-38.44-46.05) | 50 (1.84%) (-40.52-48.61) | 74 (2.51%) (-44.77-51.14) |
| HyDRA *versus* DEEPGEN | 27 (1.17%) (-37.16-41.01) | 32 (1.37%) (-38.46-42.82) | 43 (1.80%) (-41.17-45.70) | 49 (1.95%) (-42.59-46.83) | 61 (2.42%) (-45.74-49.73) | 76 (2.53%) (-48.14-50.37) |
| DEEPGEN *versus* Hivmmer | 34 (1.46%) (-43.41-38.67) | 39 (1.66%) (-45.05-39.83) | 51 (2.12%) (-47.39-41.58) | 61 (2.42%) (-50.17-43.65) | 71 (2.61%) (-54.16-47.83) | 91 (3.01%) (-57.17-50.87) |
| DEEPGEN *versus* PASeq | 33 (1.43%) (-44.66-40.79) | 38 (1.63%) (-46.82-42.58) | 47 (1.97%) (-48.53-44.19) | 59 (2.35%) (-51.66-47.76) | 70 (2.59%) (-52.70-50.15) | 102 (3.43%) (-57.13-58.01) |

**Supplementary Table S4.** The number of discordances between the analysis pipelines according to the variant detection threshold. Data from one of the six participating labs were removed from outlier analysis because the results from one pipeline were missing. In this case, there were 47 data sets as opposed to 57 (see methods). For each agreement analysis between two different pipelines, the number of discordances was calculated by counting the number of discordances outside the 95% confidence intervals, n (%) is the percentage of discordances out of the total number AAVs between two pipelines, and 95% confidence intervals were calculated using Bland-Altman analysis.

| **%frequency threshold** | **Mean ± SEM** | **P<0.05** |
| --- | --- | --- |
| ≥ 20% | 25.4 ± 1.64 |  |
| ≥ 15% | 29.7 ± 1.78 | 0.0929^a^ |
| ≥ 10% | 39.3 ± 2.55 | 0.0064^b^ |
| ≥ 5% | 52.5 ± 2.67 | 0.0022^c^ |
| ≥ 2% | 67.0 ± 3.46 | 0.0038^d^ |
| ≥ 1% | 89.4 ± 4.19 | 0.0006^e^ |

**Supplementary Table S5.** Comparison of the average number of discordances amongst the pipelines between different thresholds. Results from an unpaired 2-tailed t-test. Comparison of the number of discordances between ^a^20% and 15% threshold; ^b^15% and 10% threshold; ^c^10% and 5% threshold; ^d^5% and 2% threshold and ^e^2% and 1 % threshold.

A)


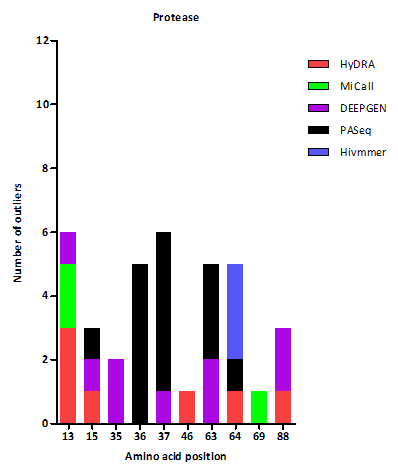

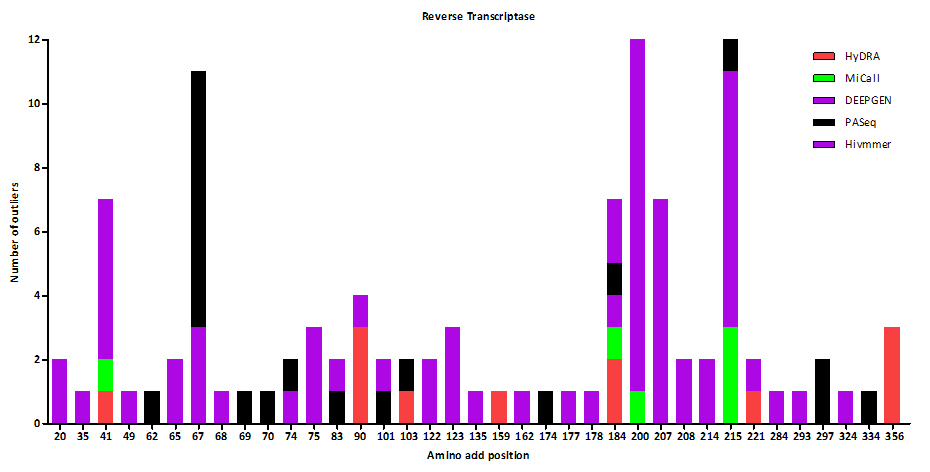

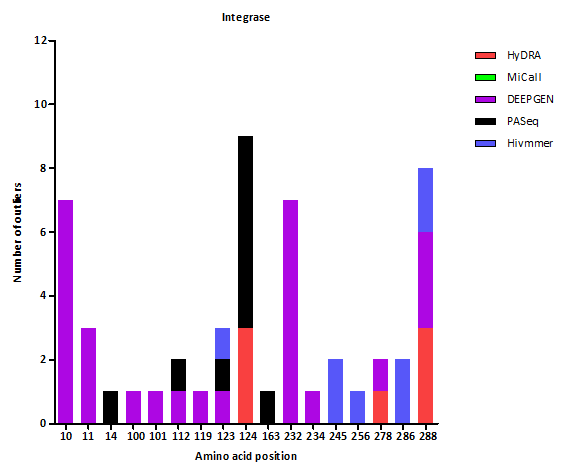


B)


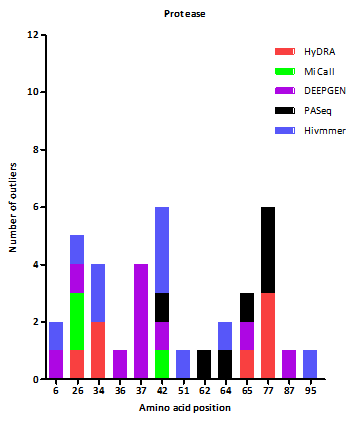

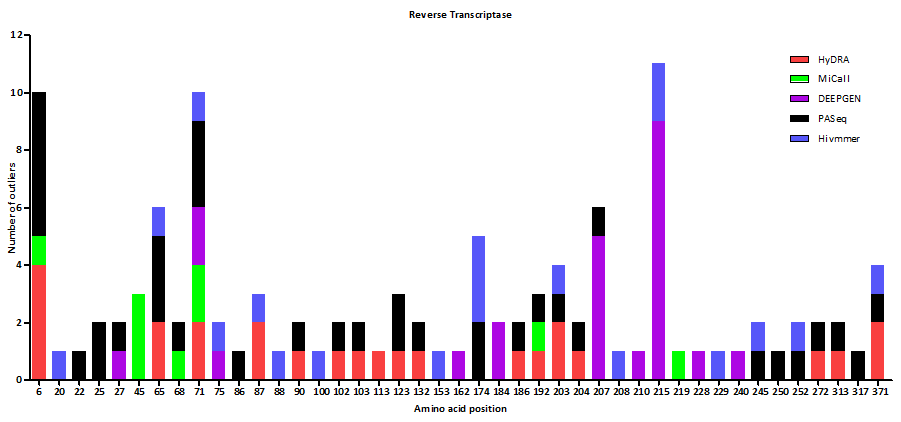

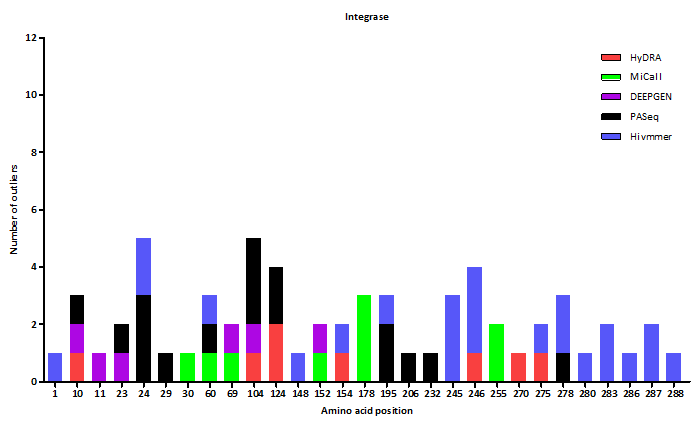


**Supplementary Figure S3.** Number of AAV outliers found in each pipeline at AAV frequencies >20% and at <20%. Thresholds for outliers were arbitrarily determined to be twice the %CV median for each defined %AAV frequency range and were calculated to be %CV ≤1%, ≤ 3%, ≤ 5%, ≤7%, ≤ 10%, ≤ 12%, ≤ 20%, ≤ 24% for AAV frequencies at ≥90%, 70%-90%, 50%-70%, 30%-50%, 20%-30%, 10%-20%, 2%-10% and 1%-2% respectively (see materials and methods). The total number of outliers found at A) >20% and B) <20% were then plotted at each amino acid position for HIV protease, reverse transcriptase and integrase genes.

|  |  |  | **% Detected^a^** | | | | |
| --- | --- | --- | --- | --- | --- | --- | --- |
| **Mutation** | **Number of data sets present** | **% AAV frequency range** | **Hydra** | **Micall** | **DEEPGEN** | **PASeq.org** | **Hivmmer** |
| **IN D10E** | 42 | > 88.54 | 100 | 100 | **83.33** | 100 | 100 |
| **IN D288N** | 4 | >98 | **50** | 100 | 100 | 100 | **50** |
|  | 1 | 6.56-7.8 | 100 | 100 | 100 | 100 | **0** |
| **PR S37A** | 4 | 15.75-9.79 | 100 | 100 | **75^b^** | 100 | 100 |
| **PR S37D** | 6 | >98 | 100 | 100 | 100 | **33^c^** | 100 |
|  | 1 | 3.03-3.43 | 100 | 100 | 100 | 100 | 100 |
| **RT D67N** | 13 | 99.72-83.72 | 100 | 100 | **100^d^** | **92.31^e^** | 100 |
|  | 2 | 40.65-76.14 | 100 | 100 | **100^f^** | **0** | 100 |
| **RT T200A** | 4 | 17.29-34.08 | 100 | 100 | **100^g^** | 100 | 100 |
| **RT Q207N** | 4 | 19.9-32.24 | 100 | 100 | **100^h^** | 100 | 100 |

**Supplementary Table S6.** Summary of AAV positions that are favored for outliers.

^a^Considered not detected if AAV frequency <1%

^b^DEEPGEN AAV frequency range is 1.02%–1.40% and had 8 extra mutations at frequency >5% (range 5.00%-24.65%)

^c^PASeq AAV frequency range is 1.065–4.98%

^d^DEEPGEN had one AAV frequency detected at 3.03%

^e^PASeq had 3 AAV frequencies detected at 1.01%, 4.12% and 8.48% respectively

^f^DEEPGEN had one AAV frequency detected at 18.63%

^g^DEEPGEN detected all 4 AAVs at 2.97%-6.71% and had 5 extra AAVs at frequencies at 14.14%-19.17%

^h^DEEPGEN frequency range is 2.97%–5.56% and has 10 extra AAVs at frequencies at 5.54%–21.64%
